# External Support of Autologous Internal Jugular Vein Grafts with FRAME Mesh in a Porcine Carotid Artery Model

**DOI:** 10.3390/biomedicines12061335

**Published:** 2024-06-16

**Authors:** Jaroslav Chlupac, Jan Frank, David Sedmera, Ondrej Fabian, Zuzana Simunkova, Iveta Mrazova, Tomas Novak, Zdenka Vanourková, Oldrich Benada, Zdenek Pulda, Theodor Adla, Martin Kveton, Alena Lodererova, Ludek Voska, Jan Pirk, Jiri Fronek

**Affiliations:** 1 Transplantation Surgery Department, Institute for Clinical and Experimental Medicine (IKEM), Videnska 1958/9, 140 21 Prague, Czech Republic; jan.frank@ikem.cz (J.F.); tomas.novak@lfmotol.cuni.cz (T.N.); jiri.fronek@ikem.cz (J.F.); 2 Department of Anatomy, Second Faculty of Medicine, Charles University, V Uvalu 84, 150 06 Prague, Czech Republic; 3 Institute of Anatomy, First Faculty of Medicine, Charles University, U Nemocnice 3, Praha 2, 128 00 Prague, Czech Republic; david.sedmera@lf1.cuni.cz; 4 Clinical and Transplant Pathology Centre, Institute for Clinical and Experimental Medicine (IKEM), Videnska 1958/9, 140 21 Prague, Czech Republic; ondrej.fabian@ikem.cz (O.F.); martin.kveton@ikem.cz (M.K.); alena.lodererova@ikem.cz (A.L.); ludek.voska@ikem.cz (L.V.); 5 Department of Pathology and Molecular Medicine, Third Faculty of Medicine, Charles University, and Thomayer University Hospital, Ruska 87, 100 00 Prague, Czech Republic; 6 Experimental Medicine Centre, Institute for Clinical and Experimental Medicine (IKEM), Videnska 1958/9, 140 21 Prague, Czech Republic; zuzana.simunkova@ikem.cz (Z.S.); iveta.mrazova@ikem.cz (I.M.); zdenka.vanourkova@ikem.cz (Z.V.); 7 Laboratory of Molecular Structure Characterization, Institute of Microbiology of the Czech Academy of Sciences, Vídeňská 1083, 142 00 Prague, Czech Republic; benada@biomed.cas.cz; 8 Department of Imaging Methods, Institute for Clinical and Experimental Medicine (IKEM), Videnska 1958/9, 140 21 Prague, Czech Republic; zdpu@seznam.cz (Z.P.); theodor.adla@ikem.cz (T.A.); 9 Third Faculty of Medicine, Charles University, Ruska 87, 100 00 Prague, Czech Republic; 10 Cardiovascular Surgery Department, Institute for Clinical and Experimental Medicine (IKEM), Videnska 1958/9, 140 21 Prague, Czech Republic; jan.pirk@ikem.cz; 11 First Surgical Clinic, First Faculty of Medicine, Charles University, U Nemocnice 499/2, 128 08 Prague, Czech Republic

**Keywords:** blood vessel prosthesis, autologous vein graft, external stent, carotid artery, pig

## Abstract

Background: Autologous vein grafts are widely used for bypass procedures in cardiovascular surgery. However, these grafts are susceptible to failure due to vein graft disease. Our study aimed to evaluate the impact of the latest-generation FRAME external support on vein graft remodeling in a preclinical model. Methods: We performed autologous internal jugular vein interposition grafting in porcine carotid arteries for one month. Four grafts were supported with a FRAME mesh, while seven unsupported grafts served as controls. The conduits were examined through flowmetry, angiography, macroscopy, and microscopy. Results: The one-month patency rate of FRAME-supported grafts was 100% (4/4), whereas that of unsupported controls was 43% (3/7, Log-rank *p* = 0.071). On explant angiography, FRAME grafts exhibited significantly more areas with no or mild stenosis (9/12) compared to control grafts (3/21, *p* = 0.0009). Blood flow at explantation was higher in the FRAME grafts (145 ± 51 mL/min) than in the controls (46 ± 85 mL/min, *p* = 0.066). Area and thickness of neo-intimal hyperplasia (NIH) at proximal anastomoses were similar for the FRAME and the control groups: 5.79 ± 1.38 versus 6.94 ± 1.10 mm^2^, respectively (*p* = 0.558) and 480 ± 95 vs. 587 ± 52 μm^2^/μm, respectively (*p* = 0.401). However, in the midgraft portions, the NIH area and thickness were significantly lower in the FRAME group than in the control group: 3.73 ± 0.64 vs. 6.27 ± 0.64 mm^2^, respectively (*p* = 0.022) and 258 ± 49 vs. 518 ± 36 μm^2^/μm, respectively (*p* = 0.0002). Conclusions: In our porcine model, the external mesh FRAME improved the patency of vein-to-carotid artery grafts and protected them from stenosis, particularly in the mid regions. The midgraft neo-intimal hyperplasia was two-fold thinner in the meshed grafts than in the controls.

## 1. Introduction

Cardiovascular disease is a leading cause of morbidity and mortality worldwide. Bypass grafting is a widely used surgical technique to treat atherosclerotic occlusive or aneurysmal disease [1]. Autologous arterial conduits, such as the internal thoracic artery or the radial artery, are considered the most durable grafts for cardiac bypass procedures. These arterial grafts exhibit an excellent 10-year patency rate of 90% [2]; however, their use is constrained by limited availability.

Autologous saphenous vein graft (SVG) is the most frequently utilized conduit for coronary (CABG) and peripheral artery bypass grafting (PABG). The saphenous vein is a superficial, non-essential vein that can be harvested from the patient’s leg(s). It is then implanted as a small-caliber (≤6 mm) arterial bypass or substitution on the heart or extremities. However, the key limitation is a high failure rate due to vein graft disease (VGD). The patency rate of SVGs drops to 50% by 10 years in both cardiac and peripheral bypass applications [1,3].

The VGD occurs because the vein, originally situated in a low-flow and low-pressure venous environment, undergoes a harvest injury and is then transplanted into the arterial circulation, which has high-flow and high-pressure conditions. These conditions increase shear stress and wall tension on the graft, leading to compensatory graft dilation and wall thickening, a process known as neo-intimal hyperplasia (NIH). NIH is characterized by the proliferation and migration of vascular smooth muscle cells (VSMCs) from the media to denuded and dysfunctional intima accompanied by the deposition of extracellular matrix. NIH comes along as diffuse wall thickening and/or focal lumen irregularities causing flow disturbances. NIH further predisposes the graft to thrombosis and accelerated atherosclerosis [3]. Such negative remodeling may result in vessel stenosis and occlusion. Early graft failures within one month post surgery are attributed to technical factors, inadequate conduit quality, poor run-off, and acute thrombosis. NIH is a primary cause of mid-term failures occurring from 1 month onward to 1–2 years postoperatively. Graft atherosclerosis is responsible for late events [4].

Currently, the only proven methods to prevent VGD involve the “no-touch” vein harvesting technique [5], lipid-lowering therapies, and antiplatelet agents [1]. Several other strategies have been explored to combat VGD, including modified surgical techniques, preservation solutions, topical ex vivo pretreatments, pharmacological interventions, gene manipulations, and external stenting [1,4].

The VGD is considered biomechanical in nature [6], justifying the use of external stents as an outer layer that protects vein grafts in arterial circulation [6]. External mesh devices influence venous wall remodeling post-arterial grafting in several ways. They reduce wall tension and prevent non-uniform dilation, thereby improving lumen uniformity and flow patterns. Furthermore, these supports facilitate adventitial neovascularization and redirect VSMC migration outwards through a reverse chemotactic gradient [7]. These actions mitigate wall thickening and suppress the formation of NIH and the development of graft atherosclerosis.

External stenting has shown considerable promise in preclinical [3,8] and, to some extent, in clinical studies [9,10] (for a review, see [6,11,12]). Most clinical trials have been conducted on CABG surgery [13]; however, reports on PABG surgery are limited [14,15].

The aim of our in vivo animal study was to investigate the effect of a current-generation FRAME external support device, which is intended for peripheral vascular surgery, on patency and neointima formation of autologous internal jugular vein interposition grafts that were implanted in porcine carotid arteries. Four weeks after implantation, we found that supported grafts had lower occlusion and stenosis rates than their control unsupported counterparts.

## 2. Materials and Methods

### 2.1. Surgery

#### 2.1.1. Implantation

Our porcine animal model with respect to carotid artery implantations has previously been described; these descriptions would include anesthesia and surgical considerations [16]. With regards to the handling of the pigs, we implemented standard means of general anesthesia (GA), analgesia, and peri-operative care. The average weight of the female domestic pigs (*sus scrofa domesticus*) at implantation was 42 ± 4 kg.

We carried out operative bilateral common carotid artery exposure with two neck incisions under sterile conditions in a supine position. Using the no-touch technique (albeit not exclusively), the internal jugular vein (IJV), which is anatomically located along the carotid artery, was carefully retrieved and gently flushed with (and stored in) warm heparinized saline. In order to identify and suture potential leaks, we applied routine gentle surgical distension with a cannula and a syringe (without pressure monitoring).

The vein was harvested unilaterally from the right side in Pigs 1 and 2 and divided into two halves, i.e., one graft each for the right and left side. In Pigs 3 and 4, the veins were retrieved bilaterally and implanted in carotid arteries on the ipsilateral sides. We consider bilateral retrieval of the IJV safe and well tolerated in swine since blood outflow from the head may be secured via external jugular veins much larger (8–9 mm) than the retrieved internal jugulars (4–4.5 mm) [17]. In addition, the diameter of the IJV grafts matched that of the carotid arteries.

We also implanted autologous IJV grafts in right-sided carotids and autologous IJV patch grafts in left-sided carotids in three additional pigs in another experiment (Pigs 5–7). We shared the right-sided implants as controls in the current study to reduce the number of animals within the 3R principle (reduction, replacement, and refinement). 

After full heparinization (200 IU/kg initially plus redosing according to activated clotting time), a ~2–3 cm-long segment of the cross-clamped common carotid artery was resected and replaced with an autologous vein interposition graft, which was implanted under optical magnification using end-to-end anastomoses with a running polypropylene 7/0 suture. The tubular FRAME mesh was threaded over the vein grafts upon completion of the proximal anastomosis and prior to suturing the distal anastomosis. The grafts were oriented in a reversed fashion due to valves enabling one-way blood flow in veins. The implanted grafts eventually needed to be longer than the excised carotid segments due to physiological retraction of the proximal and distal arterial stumps. Finally, the incisions were closed in layers, and the animals were allowed to recover. Each animal was given 100 mg of aspirin one day prior to surgery and thereafter received the same dose daily.

**Figure 1:**
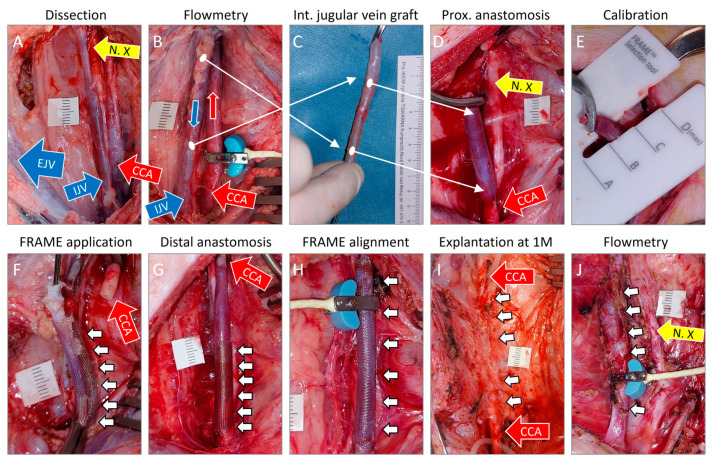
Implantation of a FRAME-supported internal jugular vein interposition graft in a porcine carotid artery. (**A**)—Dissection of a common carotid artery (CCA, red arrow) and an internal jugular vein (IJV, smaller blue arrow). A larger external jugular vein was also visible (EJV, larger blue arrow). N. X—vagal nerve, tenth cranial nerve, yellow arrow. (**B**)—A flowmetry probe was placed around the native carotid artery to measure blood flow volume prior to graft implantation. (**C**)—Retrieved reversed internal jugular vein graft. (**D**)—Completion of proximal anastomosis in an end-to-end fashion (red arrow). (**E**)—Calibration and selection of FRAME mesh diameter on a pressurized vein graft. (**F**)—Application of the FRAME mesh (white arrows) over a non-pressurized vein graft. The distal stump of the carotid artery is visible (red arrow). (**G**)—Completion of distal anastomosis in an end-to-end fashion (red arrow). The FRAME device was pushed proximally (white arrows). (**H**)—Alignment of the FRAME mesh (white arrows) over the entire graft. A flowmetry probe was placed around the FRAME-supported vein graft to measure blood flow volume immediately after implantation. (**I**)—Dissection of the graft from postoperative fibrous adhesions (white arrows) during the explantation procedure carried out one month (M) post implantation. The adjacent native carotid artery was encircled with rubber loops placed proximally and distally to the graft location (red arrows). (**J**)—Flowmetry at explantation on a surgically exposed graft.

The FRAME device was purchased from Vascular Graft Solutions (VGS, Tel Aviv, Israel; locally traded by CARDION Ltd., Brno, Czech Republic). This is a flexible, kink-resistant, braided, metal (chromium-cobalt alloy) external mesh support apparatus used for vein grafts in peripheral (i.e., non-cardiac) bypass and reconstruction procedures. The device features axial plasticity as well as radial elasticity and is supposed to mitigate the pathological remodeling of vein grafts. Product models A, B, C, and D are intended for vein graft diameters of 3.5–4.5 mm, 4.6–5.5 mm, 5.6–6.5 mm, and 6.6–8.0 mm, respectively [18]. Throughout our study, we used the model B diameter based on intraoperative calibration. Additionally, the company produces two other devices (not utilized in our study): the FRAME FR device intended for support of arteriovenous fistulas for hemodialysis access; and the VEST device used for coronary artery vein bypass grafts. No fixation of the external stents is required. Our procedure is presented in Figure 1.

In Pig 1, we deployed the FRAME support mesh over the vein grafts bilaterally, i.e., both on the right and left sides. In Pig 2, we implanted bare unsupported vein grafts bilaterally as controls. In Pigs 3 and 4, the FRAME support device was applied on the right-sided grafts, while the left sides comprised unsupported control grafts. In this manner, Pigs 3 and 4 served as their own controls. A list of all implanted grafts is presented in Table 1. The mean graft length was somewhat greater in the FRAME group (4.6 ± 0.8 cm) than in the control group (3.8 ± 1.1 cm); however, the difference was not statistically significant (*p* = 0.227).

#### 2.1.2. Flowmetry

Blood flow volume was measured using a flow probe (Transonic, ADInstruments, Oxford, UK) in the following manners and times periods: (1) on the surgically exposed native artery during the implantation procedure prior to grafting; (2) during the implantation procedure immediately after vein grafting; and (3) during the explantation procedure (one month post implantation) immediately after angiography, when the graft was dissected from postoperative adhesions. Before carrying out flow measurements, we bathed the wounds in warm saline for 10–20 min in an attempt to relax the graft spasm brought on by surgical manipulation. The highest measured flow rate was taken into account. We are aware that flowmetry is burdened by interindividual variability and may be influenced by vasospasm (see flow values before and after implantations in the Results Section 3.1.2. Flowmetry). Therefore, a more precise and gold-standard evaluation of patency is through angiograms of undissected vessels.

#### 2.1.3. Angiography

After a period of 1 month, i.e., 28 days, the pigs underwent angiographic examination and graft explantation under a second GA. The average body weight of the animals at explantation was 58 ± 5 kg. Selective carotid angiography (X-ray mobile C-arm Ziehm Vision FD, Ziehm Imaging, Ltd., Nuremberg, Germany) with an iodinated contrast agent (Optiray, Guerbet, Princeton, NJ, USA) was carried out using the femoral artery access according to the Seldinger technique. Each graft was arbitrarily divided into three portions: proximal anastomosis, mid-graft, and distal anastomosis; this amounted to a total of 12 areas (4 grafts × 3 portions) in the FRAME and 21 areas (7 grafts × 3 portions) in the control group. The degree of eventual stenosis in each graft area was calculated according to the North American Symptomatic Carotid Endarterectomy Trial (NASCET) formula, i.e., [1 − (G/C) ] × 100, wherein G represents the diameter of the investigated graft portion and C represents the diameter of the adjacent native carotid artery [19]. We carried out anterior-posterior, right lateral, and left lateral projections at an angle of 30°. Therefore, narrowing (expressed as percentage) was calculated as a mean of these three values. A narrowing of ≤40%, 41–60%, and >60% were considered mild, moderate, and severe cases of stenosis, respectively.

#### 2.1.4. Explantation

Immediately following the angiographic examinations, we carefully dissected the implants, carried out flowmetry measurements on exposed grafts, and explanted them along with parts of native carotid arteries. Heparin was not administered during the explantation procedure. The retrieved samples were gently flushed with warm saline, cross-sectioned at equidistant points along the entire graft, and photographed. The animals were then euthanized with an overdose of thiopental and potassium.

### 2.2. Macroscopic Examinations

**Figure 2:**
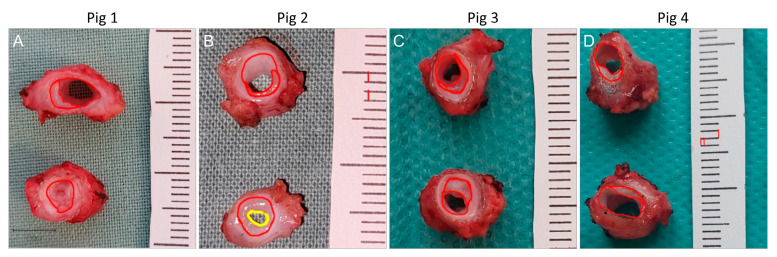
Measurement of neo-intimal hyperplasia (NIH) area and thickness using zoomed macrographs of cross-sectioned explants in Pigs 1, 2, 3, and 4 ((**A**), (**B**), (**C**), and (**D**), respectively). Average NIH thickness (μm^2^/μm) was calculated as the NIH area (μm^2^) divided by the original lumen circumference (μm).

All cross-section photo-macrographs were zoomed-in and examined for the development of luminal neo-intima. We used QuPath open-source software (version 0.3.0) to analyze macrograph images [20]. Average NIH thickness (μm^2^/μm) was calculated as the NIH area (μm^2^) divided by the presumed (original) lumen circumference (μm) [19]. The mean values were calculated from all sections of each graft within a specific group. We used this formula uniformly with all possible NIH events: circular, semicircular (i.e., not covering the entire lumen circumference), and even with total graft occlusions (Figure 2). We observed NIH in all cases, with the exception of the left-sided midgraft in Pig 1; we therefore took a zero value into account in this case.

Near occlusion and total graft occlusions that occurred in the control group can induce atrophy, i.e., shrinkage of the graft diameter. As such, the NIH area divided by the original lumen circumference may give lower values. Despite this, the outcomes regarding NIH formation were more favorable in the FRAME group than in the control group. In contrast, other studies did not include occluded grafts in their analysis [21].

NIH values were calculated separately for proximal anastomoses and midgraft sections. Sufficient NIH measurements for distal anastomoses could not be acquired due to technical reasons: three of the four distal anastomoses in the FRAME group and two in the control group were not cross-sectioned but rather cut open longitudinally, which impeded analysis more so than with the other graft portions. Distal anastomotic regions, however, were evaluated angiographically.

We could not process the supported grafts for histological examinations because of the adhering FRAME mesh. The presence of any metal material precludes common histological sectioning. The required technology and equipment, which include methyl-methacrylate resin embedding as well as appropriate tungsten carbide blade for cutting [7,22], were not available to us [23].

We are aware of the potential inaccuracy in measuring NIH parameters using macrographs as opposed to histological images, which produce more precise measurements. Similar inaccuracies, however, were most likely present for the FRAME and control groups. That is why we considered detailed macroscopic image analysis sufficient for group comparisons. Moreover, digital analysis of macroscopic images has been reported in previous studies [22].

### 2.3. Microscopical Examinations

#### 2.3.1. Histology and Immunohistochemistry

The cross-sectioned control samples (without FRAME metal support) were emersion fixed with 4% formaldehyde (48 h), embedded in paraffin, cut into 4–5 μm thick sections, mounted on glass slides, dried and stained with hematoxylin and eosin (Merck & Co., Inc., Kenilworth, NJ, USA) and Weigert van Gieson and Resorcin-fuchsin for elastic fibers (proprietary method). We carried out immunostaining for the expression of alpha-smooth muscle actin (primary antibody: REF: 202M, Cell Marque Corp., a part of MilliporeSigma, Rocklin, CA, USA; secondary antibody: provided as part of the UltraView Universal DAB Detection Kit, REF: 760–500, Ventana Medical Systems, Inc., Roche Group, Tucson, AZ, USA), endothelial marker CD31 (primary antibody: REF: NB100-2284, Novus Biologicals, LLC, Centennial, CO, USA; secondary antibody: REF: BA-1000, Vector Laboratories, Inc., Burlingame, CA, USA), and endothelial marker ERG (primary antibody: REF: 434R, Cell Marque Corp., a part of MilliporeSigma, Rocklin, CA, USA; secondary antibody: provided as part of the OptiView DAB IHC Detection Kit, REF: 760–700, Ventana Medical Systems, Inc., Roche Group, Tucson, AZ, USA). The slides were viewed and scanned with an Olympus VS110-S5 Slide Scanner (Olympus, Hamburg, Germany).

We used QuPath open-source software (version 0.3.0) for digital pathology image analysis [20] and measured NIH area and thickness in multiple midgraft histological sections of the control grafts. The NIH thickness was calculated in a manner identical to that of the macrograph method, i.e., the NIH area divided by the original lumen circumference. These parameters can be measured precisely using histology images: the NIH area was defined as any neo-tissue luminal from internal elastic lamina, and the presumed (original) lumen circumference was delineated by this internal elastic lamina. The neo-tissue abluminal from external elastic lamina was termed neo-adventitia.

#### 2.3.2. Macro Photography

Macro photographs were taken under an Olympus SZX125 dissecting microscope using, a 1.0× objective, and DP 74 CCD camera (Olympus, Japan).

#### 2.3.3. Confocal Microscopy

Confocal imaging was performed following whole mount staining, as described recently [24], with anti-von Willebrand factor antibody (1:50, Sigma #3520, Sigma-Aldrich, Saint Louis, MO, USA), detected with Cy5 secondary antibody (Jackson ImmunoResearch, Cambridgeshire, UK) to label the endothelial cells. The instrument used was an Olympus BX61 upright microscope fitted with an Olympus Fluoview FV1000 confocal system (Olympus, Hamburg, Germany). The stained specimens underwent clearing in CUBIC for 24 h, as described in references [25,26], and were then pinned to the bottom of a deep Sylgard-coated Petri dish for imaging. Imaging utilized a 2× 0.14 NA dry and a 10× 0.6 NA multi-immersion objectives with appropriate excitation and emission settings. A 25× 1.0 NA multi-immersion objective was used for high-power views, allowing for up to 1 µm z-resolution.

#### 2.3.4. Scanning Electron Microscopy (SEM)

SEM was carried out using an established protocol [27]. The fixed samples underwent extensive washing in phosphate-buffered saline (1× PBS, pH 7.2) and were postfixed in a buffered solution of 1% OsO_4_ for one hour at room temperature. Following another round of extensive washing with 1× PBS, the samples were dehydrated in a graded series of alcohols (25%, 50%, 70%, 80%, 96%, 100%, and 100%) and subsequently dried in a K850 Critical Point Dryer (Quorum Technologies Ltd., Ringmer, UK). The dried samples were then mounted onto standard aluminum SEM stubs and sputter-coated with a 3 nm layer of platinum using a high-resolution Q150T Turbo-Pumped Sputter Coater (Quorum Technologies Ltd., Ringmer, UK). For the final analysis, the samples were examined under an FEI Nova NanoSEM 450 scanning electron microscope (FEI, Brno, Czech Republic) employing an SED detector at a voltage of 5 kV.

### 2.4. Statistical Analysis

Continuous variables with normal distribution were expressed as mean and standard deviation (SD). NIH values are expressed as the mean and standard error of the mean (SE). A two-tailed Student’s *t*-test was used to compare two data sets, while a one-way analysis of variance (ANOVA, Newman–Keuls test) was implemented for three data sets, and finally, the log-rank (Mantel-Cox) test was used for survival (patency) analysis. Categorical variables were presented as positive and negative observations (expressed as percentages) and compared using a two-tailed Fisher’s exact test. Data were computed in Microsoft Excel (version 2108) spreadsheets. We utilized GraphPad Prism software (version 5.03, 2009) for statistical evaluations. A *p*-value of ≤0.05 was considered statistically significant.

## 3. Results

### 3.1. Surgery

#### 3.1.1. Implantation

**Figure 3:**
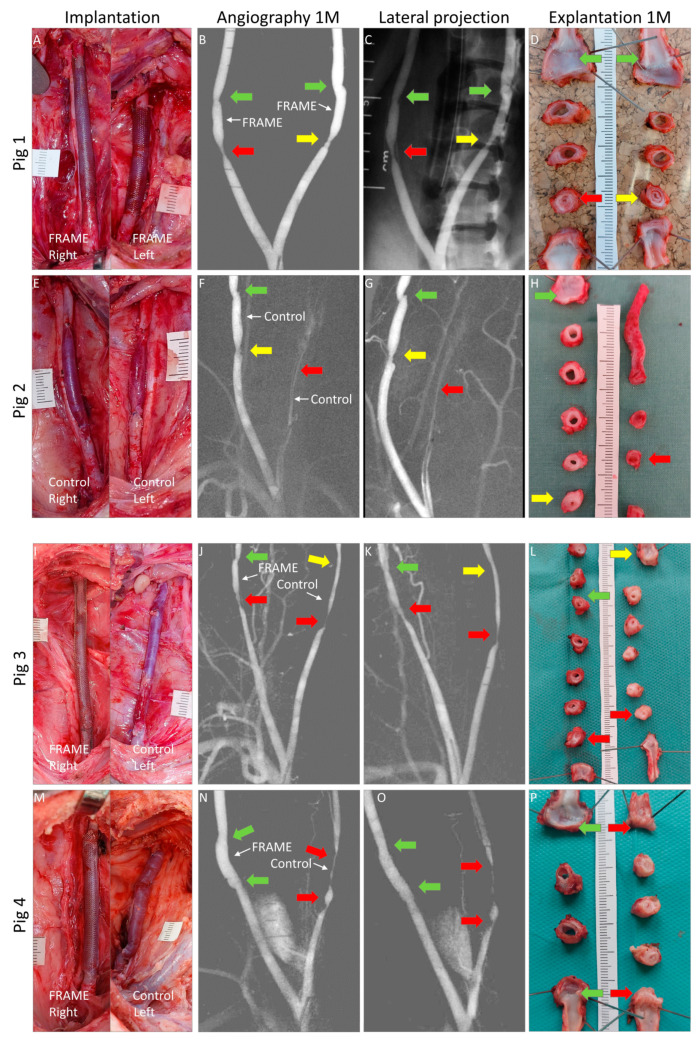
Implantation, pre-explantation angiography, and explantation of autologous internal jugular vein interposition grafts in porcine carotid arteries of Pigs 1 (**A**–**D**), 2 (**E**–**H**), 3 (**I**–**L**), and 4 (**M**–**P**). We implanted grafts supported with FRAME mesh as well as unsupported grafts as controls. Macroscopic views of the grafts after declamping and hemostasis during implantations are shown in the left-hand column. Selective carotid angiograms performed from femoral access at 1 month (1 M) post implantation are shown in the middle two columns (anterior-posterior and lateral projections). Gross appearances of cross-sectioned explants at 1 M are presented in the right-hand column. Proximal and distal anastomoses are marked with arrows; green indicates no or mild stenosis (≤40%), yellow moderate (41–60%), and red severe stenosis (>60%) or occlusion.

**Figure 4:**
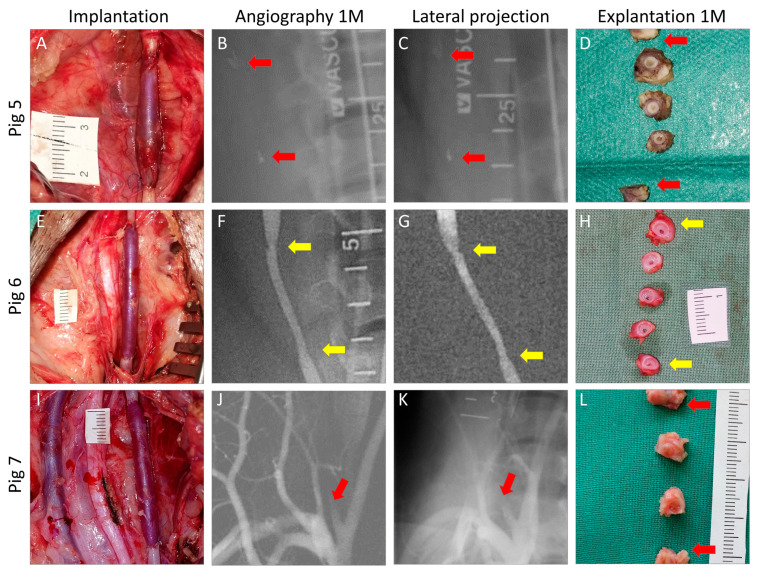
Implantation, pre-explantation angiography, and explantation of autologous internal jugular vein grafts in porcine carotid arteries of Pigs 5 (**A**–**D**), 6 (**E**–**H**), and 7 (**I**–**L**). We implanted interposition grafts to the right-sided carotids and patch grafts to the left-sided carotids. We utilized the right-sided implants as controls to reduce the numbers of experimental animals. Macroscopic views of the grafts after declamping and hemostasis during implantations are shown in the left-hand column. Selective carotid angiograms performed from femoral access at 1 month (1 M) post implantation are shown in the middle two columns (anterior-posterior and lateral projections). Gross appearances of cross-sectioned explants at 1 M are presented in the right-hand column. Proximal and distal anastomoses are marked with arrows; green indicates no or mild stenosis (≤40%), yellow moderate (41–60%), and red severe stenosis (>60%) or occlusion. (**B**,**C**)—red arrows point at metal clips, which indicate boundaries of the occluded grafts. (**J**,**K**)—red arrows point at proximal stumps of occluded carotid arteries.

The mean surgery time was 152 ± 28 min. There were no significant peri-operative or post-operative adverse events. The mean carotid artery clamping time was 40 ± 8 min in the FRAME group and 33 ± 6 min in the control group (*p* = 0.116). The slightly longer time for the FRAME group was statistically non-significant (n.s.), which indicates favorable and swift surgical handling when deploying the FRAME device. Intra-operative views of implanted constructs, pre-explant angiograms, and macrographs of cross-sections explanted at 1 month post implantation are shown in Figure 3. Views of the additional three control grafts are shown in Figure 4.

#### 3.1.2. Flowmetry

Flowmetry results and mean arterial pressures (MAPs) at three time points: native carotid artery before graft implantation, after graft implantation, and explantation at one month (i.e., after 28 days), are presented in Table 2. In the FRAME group, mean blood flow dropped insignificantly after implantation, most likely due to vasospasms and graft placement per se. Differences between the three time points were not significant. At explantation, flow in the right-sided graft in Pig 1 was markedly lower than in the three remaining FRAME-supported grafts, indicating more significant stenosis in proximal anastomosis consistent with the angiograms. Flow in the remaining three FRAME-supported grafts was normal.

In the control group, mean blood flow after graft implantation dropped significantly, most likely also due to vasospasms, graft placement per se, as well as lower, albeit nonsignificant MAP. The mean flow at explantation dropped markedly, which was statistically significant in contrast to the values prior to implantation. Neither the FRAME group nor the control group exhibited any differences between MAPs.

**Figure 5:**
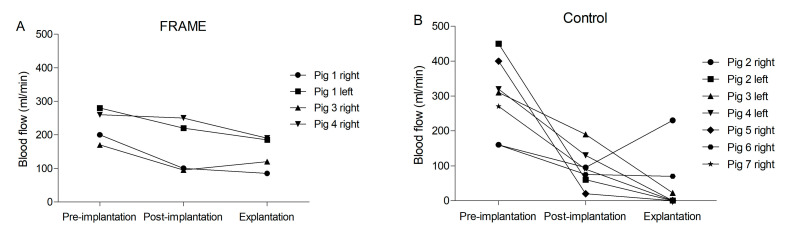
Flowmetry results in (**A**) FRAME-supported and (**B**) control groups of autologous internal jugular vein interposition grafts in porcine carotid arteries at three time points: native carotid artery prior to graft implantation, after graft implantation, and at explantation (1 month post implantation). See Table 2 for exact numbers and statistics.

There were no statistically significant differences in mean flows nor in the MAPs between the FRAME and control groups at any time point. The flow at explantation in the control group was zero in four out of the seven grafts due to graft occlusions and as low as 22 mL/min in the left-sided graft in Pig 3, indicating a failing graft. Flow in the stenotic right-sided graft in Pig 6 was subnormal. The only graft with sufficient flow in the control group was the right-sided graft in Pig 2. Flowmetry results at explantation were, therefore, generally better in the FRAME group, although narrowly missing statistical significance (*p* = 0.066) due to high variations (Figure 5).

#### 3.1.3. Angiography

**Figure 6:**
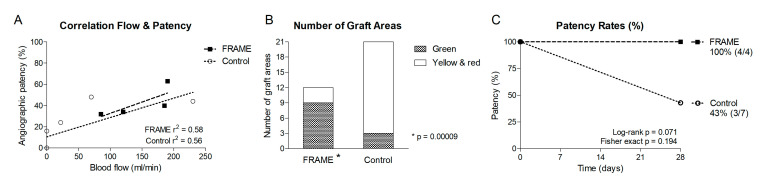
(**A**)—Correlation between blood flow and angiographic patency of FRAME-supported and control autologous internal jugular vein interposition grafts in porcine carotid arteries one month post implantation. (**B**)—Numbers of green versus yellow and red areas in the heat map from Table 3. Green indicates no or mild stenosis (≤40%), while yellow and red indicate moderate (41–60%) and severe stenosis/occlusion (>60%), respectively. Each graft was divided into three sections: proximal anastomosis, midgraft, and distal anastomosis. This provided a total of 12 areas in the FRAME group and 21 in the control group. There are significantly more green areas in the FRAME group (9/12, *p* = 0.0009) than in the control group (3/21). (**C**)—Patency rates in the FRAME-supported and control groups. The graph is a schematic representation of patency rates at one month, i.e., 28 days post implantation, since we do not know the exact times of graft occlusions, i.e., drops of the curve in the control group. The one-month patency rate was 100% (4/4) for the FRAME and 43% (3/7) for the control group. The difference fell just short of statistical significance (Log-rank *p* = 0.071, Fisher exact *p* = 0.194), most likely due to the low number of grafts.

Angiography results in the form of a heat map are summarized in Table 3. They closely correlate with flowmetry results from Table 2 and Figure 5. The correlation is shown in Figure 6A; the percentage of angiographic patency was inversely proportional to the percentage of the most severe angiographic stenosis in each graft listed in Table 3. The left-sided graft in Pig 4 appeared near occluded on the angiogram; however, we considered this graft completely occluded given the zero flow reading.

Nine out of twelve areas in the FRAME group were green, whereas only three out of twenty-one areas in the control group were green (Figure 6B), which indicates significantly more favorable angiographic results in the FRAME group (*p* = 0.0009). The one-month patency rate of FRAME-supported grafts was 100% (4/4), while that of unsupported control grafts was 43% (3/7) (Figure 6C). However, the obvious difference in patency rates narrowly missed statistical significance (Log-rank *p* = 0.071, Fisher exact *p* = 0.194), which was most likely due to the low number of grafts.

#### 3.1.4. Explantation and Macroscopical Examinations

**Figure 7:**
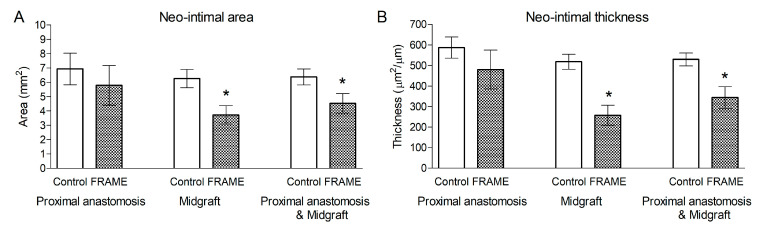
(**A**) Area and (**B**) thickness of neo-intimal hyperplasia (NIH) in FRAME-supported and control internal jugular vein interposition grafts in porcine carotid arteries at one month post implantation. Data are given as means ± standard errors of NIH area and thickness computed from a digital analysis of macrographs of serial cross-sections along the grafts. Thickness was calculated as NIH area divided by the original lumen circumference. The asterisks (*) indicate statistical significance between FRAME and control groups.

Average NIH area and thickness values calculated from macrograph analysis are given in Table 4 and in Figure 7. The NIH was less pronounced in FRAME-supported than in control grafts, albeit non-significantly in proximal anastomoses. However, in midgraft sections, the difference was statistically significant; this was also the case when proximal anastomosis and midgraft portions were calculated together, which gave an overview of the majority of the grafts. Mild eccentric semicircular non-stenosing NIH was observed in midgraft portions in three of the four FRAME-supported grafts. This type of NIH was not visible on angiograms. Notably, there was no NIH whatsoever in the left-sided midgraft of Pig 1 (Figure 3D).

We were unable to measure NIH in distal anastomoses due to technical issues. There were, however, no significant angiographic stenoses in the distal anastomoses of the FRAME group, which was in contrast to the control group (Figure 3 and Table 3). This is further indication of the overall more favorable outcomes provided by the FRAME support in terms of protection against NIH development.

### 3.2. Microscopical Examinations

#### 3.2.1. Histology and Immunohistochemistry

**Figure 8:**
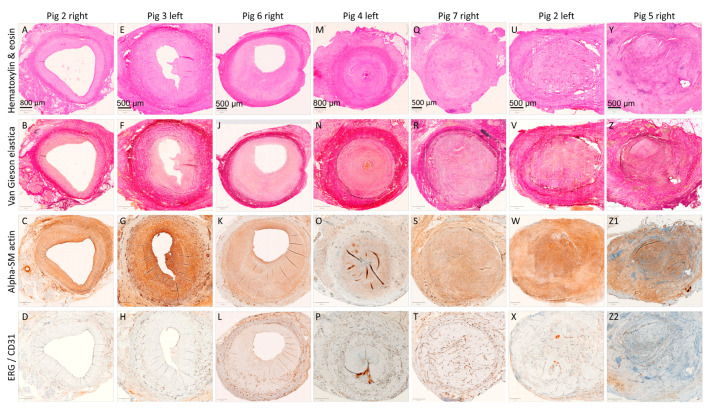
Microscopical examinations of autologous internal jugular vein interposition grafts in porcine carotid arteries at one month post implantation (control group). Representative cross-sections of the midgraft regions are shown in Pigs 2 (**A**–**D** and **U**–**X**), 3 (**E**–**H**), 4 (**M**–**P**), 5 (**Y**–**Z2**), 6 (**I**–**L**), and 7 (**Q**–**T**). The seven control samples are ordered from left to right according to degree of pathological remodeling and vein graft lesions, i.e., patent, mildly stenotic, severely stenotic, or totally occluded (see Figure 9 for more details). We performed histological staining with hematoxylin and eosin and Weigert van Gieson and Resorcin-fuchsin (elastica), as well as immunohistochemistry of alpha-smooth muscle (SM) actin and endothelial markers ERG (**D**,**L**,**P**,**X**) or CD31 (**H**,**T**,**Z2**). Magnification was 20×.

**Figure 9:**
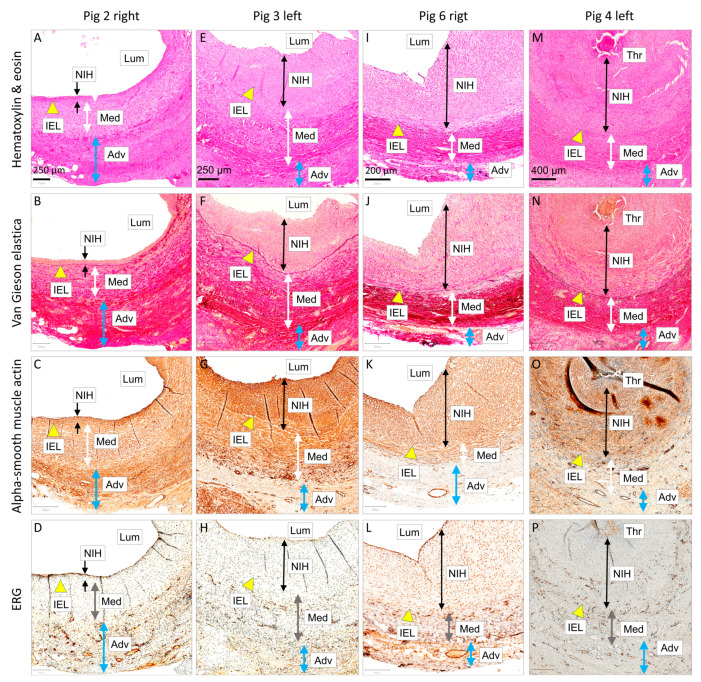
Detailed microscopical examinations of autologous internal jugular vein interposition grafts in porcine carotid arteries at one month post implantation (control group). Representative cross-sections of the midgraft regions are shown. (**A**–**D**) shows a patent graft with minimal neointimal hyperplasia (NIH), (**E**–**H**) shows a patent graft with moderate NIH, (**I**–**L**) shows a patent graft with severe NIH, and (**M**–**P**) shows a graft occluded with substantial NIH and a thrombus. We performed histological staining with hematoxylin and eosin and Weigert van Gieson and Resorcin-fuchsin (elastica), as well as immunohistochemistry of alpha-smooth muscle actin and the endothelial nuclear marker ERG. Magnification was 20×. Abbreviations: Lum—lumen, NIH—neo-intimal hyperplasia, Med—tunica media, Adv—tunica adventitia (neo-adventitia), IEL—internal elastic lamina, and Thr—thrombus.

Histology and immunostaining examinations of the unsupported control grafts are shown in Figure 8. The seven samples were lined up according to degree of pathological remodeling and vein graft lesions, i.e., patent, mildly stenotic, severely stenotic, or totally occluded. The micrographs correlate with angiography and flowmetry examinations. Four of the seven grafts became occluded (Pigs 2 left, 4 left, 5 right, and 7 right), two remained patent but developed substantial neointima (Pigs 3 left and 6 right), and only the right-sided graft in Pig 2 was wide open with insignificant NIH. Detailed microscopical examinations are shown in Figure 9. Endothelial coverage was visible in the patent grafts.

The control midgrafts, calculated from histological sections, had a mean NIH area and thickness of 4.73 ± 0.58 mm^2^ and 458 ± 45 μm^2^/μm, respectively. These values were 25% (area) and 12% (thickness) lower than those calculated from the zoomed-in photo-macrographs (6.27 ± 0.64 mm^2^ and 518 ± 36 μm^2^/μm, respectively), most likely as a consequence of tissue shrinkage during histological processing. Zilla et al. presented a similar fixation-related tissue shrinkage of 9.9% ± 3.9% [28]. We could not directly compare histological NIH values between the FRAME and control groups since we were unable to conduct histological examinations of the FRAME-supported grafts (see Materials and Methods). However, the mean NIH thickness in the FRAME midgrafts (258 ± 49 μm^2^/μm) was still significantly lower than that in the control grafts, even when utilizing two different methods of measurement: macroscopic in FRAME and histological (i.e., shrank) in control (*p* = 0.0062). It is noteworthy that other research studies have acquired and compared vascular dimensions from two different measurement methods, e.g., ultrasound and the morphometry of pressure-fixed samples [28].

#### 3.2.2. Confocal Microscopy and Scanning Electron Microscopy (SEM)

**Figure 10:**
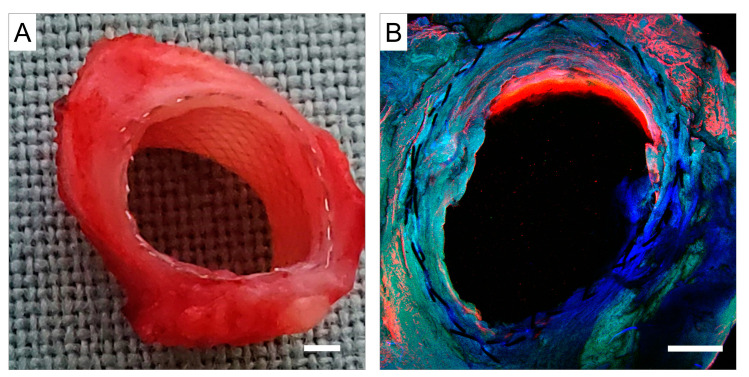
Pig 1—Representative photographs of the left-sided midgraft section at explantation (one month post implantation). (**A**)—Photo-macrograph, scale bar = 1 mm. The graft is patent, and the FRAME mesh is visible from the inside. (**B**)—Photo-micrograph, confocal microscopy, scale bar = 1 mm. The graft lumen is lined with endothelial cells (red color). Red represents staining for von Willebrand factor; blue represents 4′,6-diamidino-2-phenylindole (DAPI) counterstain for cell nuclei; and green is autofluorescence (confocal microscope Olympus Fluoview FV1000).

The macrograph and corresponding confocal micrograph of the midgraft section of the left-sided FRAME-supported graft of Pig 1 are presented in Figure 10. The graft was wide open, and there was literally no neointimal formation. Due to the graft wall being extremely delicate, the mesh contours were visible throughout its entirety. The lumen was smooth and lined with endothelial cells.

**Figure 11:**
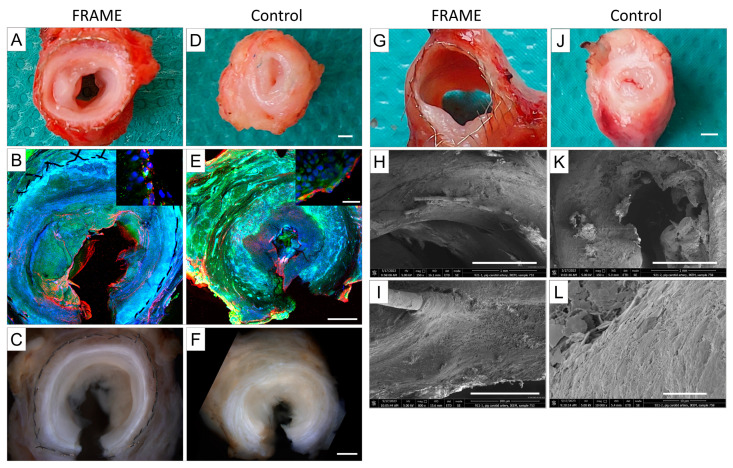
Pig 3 and Pig 4 (representative photographs at explantation, i.e., one month post implantation); (**A**–**F**), left two columns: representative photographs of proximal graft sections of Pig 3. First column (**A**–**C**): right-sided FRAME-supported graft; semicircular neo-intimal hyperplasia (NIH) causing mild stenosis is visible in the lumen. Second column (**D**–**F**): left-sided unsupported control graft; circular NIH causing severe stenosis is found in the lumen. (**A**,**D**)—photo-macrographs, scale bar = 1 mm. (**B**,**E**)—confocal micrographs, magnification 2×, scale bar = 1 mm; insets: high-power views, magnification 25×, scale bar = 50 μm. Both grafts possess endothelial cells in the lumen. Red represents staining for von Willebrand factor; blue represents 4′,6-diamidino-2-phenylindole (DAPI) counterstain for cell nuclei; and green is autofluorescence (confocal microscope Olympus Fluoview FV1000). (**C**,**F**)—macrographs from a dissecting microscope, scale bar = 1 mm. (**G**–**L**), right two columns: representative photographs of midgraft sections of Pig 4. Third column (**G**–**I**): right-sided FRAME-supported graft; semicircular NIH (no stenosis) is visible in the lumen. Fourth column (**J**–**L**): left-sided unsupported control graft; circular NIH (near occlusion) is visible in the lumen. (**G**,**J**)—photo-macrographs, scale bar = 1 mm. (**H**,**I**,**K**,**L**)—scanning electron microscopy, (**H**,**K**): scale bars = 1 mm, (**I**): scale bar = 200 μm, and (**L**): scale bar = 10 μm; FEI Nova NanoSEM 450 scanning electron microscope.

Macrographs, confocal micrographs, macrographs taken with a microscope, and SEM micrographs of the right-sided FRAME-supported grafts and left-sided bare control grafts from the same animal are shown in Figure 11. Semicircular NIH regions that brought about mild stenosis (Pig 3) and no stenosis (Pig 4) were visible in the lumen of the FRAME-supported grafts. Conversely, NIH in the form of severe circular stenosis (Pig 3) and near occlusion (Pig 4) was found in the control grafts. All grafts were lined with endothelium.

## 4. Discussion

In our in vivo animal study, we demonstrated the beneficial effects of external support on porcine autologous vein-to-carotid artery interposition grafts. Specifically, we observed improvements in patency rates and a reduction in the formation of neointimal hyperplasia compared to control group without support. Our novel findings include (1) confirming the efficacy of the latest generation external FRAME stenting device in preventing adverse remodeling in the midgraft regions of veins, and (2) utilizing a specific model involving internal jugular vein grafts interposed into the carotid artery circulation in pigs. The beneficial effect was less pronounced at the proximal anastomoses.

### 4.1. Animal Model

A recent systematic review (2023) on large animal models of vein graft hyperplasia [8] identified five studies that utilized porcine internal jugular veins as vascular graft in carotid arteries [29,30,31,32,33]. Additionally, we have found one more [34]. All these studies investigated the mechanisms or treatments of vein graft disease but did not involve the use of an external stenting device. The sole study that explored the use of peri-vascular wrap on porcine IJVs was conducted in vitro in a perfusion system [35]. The in vivo study designs and vein graft patency rates, in comparison to our study, are presented in Table 5. The studies employing interposition end-to-end grafting do not mention anastomosis beveling [29,31,34], with one exception that explicitly reported the absence of beveling [33]. Additionally, most studies have omitted details regarding intraoperative graft storage solutions [30,31,32,33]. In the gene manipulation studies, the vein grafts were specifically rinsed in the gene transfection solutions [29,34].

The published patency rates of porcine internal jugular vein-to-carotid artery grafts varied from 37.5% to 100% at various time points. One-month patency rate of the control grafts in our series was 42.9% (3/7). In analogy to our study, Quint et al. achieved a one-month-patency rate of 37.5% (3/8) in an end-to-side bypass model [32].

In comparison, several large series of pig distended saphenous vein-to-carotid artery interposition grafting demonstrated patency rates of, e.g., 60% (15/25) at one month [36] and 64% (16/25) at 22 ± 2 (range 5–37) days [37]. Interestingly, grafts made with un-distended veins harvested with a “no-touch” technique had significantly better patency rates of 89% (8/9) [21], 96% (24/25) at 16 ± 2 (range 7 to 36) days [37], or even remarkable rates of 100% (9/9) at 4 weeks [38]. Our inferior patency of the control grafts may possibly be explained by using other graft types (jugular vs. saphenous veins) [37], not strictly applying the “no-touch” harvesting technique [36], and using different animal subspecies [34].

In the majority of our FRAME midgrafts, mild semicircular eccentric not stenotic NIH was found, analogous to other studies [22]. This type of NIH, however, was not visible on angiograms and may thus be regarded as clinically insignificant. On the contrary, control midgrafts displayed NIH and negative remodeling ranging from stenotic lesions to total occlusions. Notably, there was no intimal thickening at all in the left-sided FRAME-supported midgraft in Pig 1. The contralateral right-sided graft was also supported with FRAME and developed only mild lesions. By contrast, no adverse remodeling either was found in the right-sided control graft in Pig 2. Here, the contralateral graft was also unsupported and became occluded. This occlusion, however, might have been related to technical factors or poor vein quality (insufficient diameter). It seems that FRAME functioned fully protectively in one particular animal while the support was not needed in another particular one. It has been postulated that some vein grafts adapt better to arterial conditions while others undergo progressive negative remodeling [28,39]. In humans, possible individual genetic predisposition to vein graft disease has been reported; polymorphism in the p27Kip1 gene was associated with improved peripheral vein graft patency [40].

### 4.2. Anastomosis Considerations

Instructions for Use (IFU) advise covering the entire graft with the FRAME mesh and positioning it as close as 2 mm to the anastomotic sites. In our experiment, the mesh extended a few millimeters beyond the anastomoses, as shown in Figure 1 and Figure 3. This extension is feasible in end-to-end anastomosis within interposition grafting, unlike the end-to-side anastomosis commonly used in bypass grafting. Specifically, coronary artery procedures exclusively use end-to-side bypass grafting, while both end-to-side and end-to-end configurations are employed in peripheral vascular reconstructions, including surgery on extremity arteries. In their clinical series on replacing popliteal artery aneurysms, Ciftci et al. (2021) adhered to the IFU guidelines [15]. Conversely, several experimental studies, including ours, have placed the mesh over the anastomotic regions [7,21,41,42].

Notably, anastomotic lesions in our study emerged irrespective of the FRAME device’s use, with stenoses developing more frequently at proximal than at distal anastomoses in both FRAME and control groups. Consequently, we believe that extending the mesh over the end-to-end anastomoses in our experimental setup did not adversely affect the outcomes. We further hypothesize that this placement may even offer additional protection, particularly in distal anastomoses.

From a technical standpoint, the stent should initially permit unrestricted graft expansion in response to arterial pressure. Additionally, a close fit of the external stent along the vein graft is crucial for its effective function. The stent is supposed to be designed highly porous and become incorporated into the neo-adventitia, as disruption of the adventitia can lead to vessel wall hypoxia [38]. However, the anastomotic site is generally more susceptible to NIH development due to surgical trauma from stitches, graft/artery compliance and diameter mismatches [3], and increased platelet aggregation [34]

In analogy to our findings, Zilla et al. (2011) observed manifold thicker NIH in anastomotic regions compared to midgraft regions, possibly attributed to pannus overgrowth from adjacent arteries, with this disparity being notably more pronounced in proximal anastomoses [43]. Conversely, other studies have focused solely on NIH formation in midgraft regions [22,28].

### 4.3. Existing Preclinical Data

Parsonnet et al. proposed using external support for vein grafts for the first time in 1963. They applied a knitted polypropylene tube stent over the external jugular vein to carotid artery interpose grafts in dogs to prevent dilation for up to 63 days [44]. External stents made of various nonabsorbable and absorbable, loose-fitting and tight-fitting, polymeric and metal materials (polyethylene terephthalate—Dacron, polytetrafluoroethylene—PTFE, polyethylene, polypropylene, polyester, polygalactin, polydioxan, braided or knitted nitinol, chrome-cobalt alloy) have been further tested as a treatment for vein graft disease in several animal models (murine, leporine, canine, porcine, ovine), anatomical positions (carotid, femoral, and coronary), and observational periods (usually from 4 weeks to up to 6 months); for reviews, see [6,11]. For instance, a PTFE wrap around jugular vein grafts interposed in common carotid arteries significantly reduced wall thickness, cross-sectional area, and formation of foam cells in both normocholesterolemic and hypercholesterolemic rabbits for 8 weeks [45].

Large animal models, however, have predominated, owing to vessel dimensions similar to man. In an ovine model, Ramachandra et al. (2022) implanted four external jugular-to-carotid artery grafts that were externally wrapped with a biodegradable, braided Vicril-Rapide scaffold. All of the wrapped grafts remained patent at four months; meanwhile, of the four control grafts, one developed an aneurysm, one exhibited severe stenosis, and two remained patent with no signs of stenosis [41].

The research group of Angelini et al. showed numerous short-term and long-term beneficial effects of polyester and polygalactin external stents on suppression of neointimal hyperplasia and early atherosclerotic events in a model of pig saphenous vein-to-carotid artery interposition grafting [21,38,46,47,48,49,50,51].

The group of Zilla et al. demonstrated positive effects of braided and knitted nitinol stents on various vessel dimensions in saphenous veins implanted as femoral interposition or coronary bypass grafts in nonhuman primates—senescent Chacma baboons [22,28,42,43,52,53,54] whose anatomy and vascular healing responses are closest to man [55]. They even showed that extreme constriction led to almost absent neointima formation in femoral grafts [53]. Based on different flow hemodynamics, shear stress patterns, and target artery-to-graft caliber mismatch degrees, they found distinctly more intimal hyperplasia in coronary than in femoral vein grafts [28]. Thus, the inhibitory effect of nitinol stenting on neointimal tissue formation was still significant in coronary but less pronounced than in infra-inguinal femoral grafts using the same baboon model [22]. Notwithstanding, they declared that milder coronary artery/saphenous vein graft diameter mismatch in humans would make the effect of mesh likely to be more pronounced when clinically used [22].

Preclinical testing showed considerable promise; nonetheless, due to a range of technical or methodological issues, none of the external support frameworks were adopted into routine surgical procedures [56].

Similar types of external stents as those in our study have been evaluated in the following two preclinical studies in sheep. Ben-gal et al. (2013) proved the Fluent external stent device (braided cobalt-chromium-nickel-molybdenum-iron alloy, VGS, Tel Aviv, Israel) to be efficacious in reducing saphenous vein graft irregularity and intimal hyperplasia and improving graft patency rate in an ovine case-controlled model (n = 10) of cardiac revascularization over a period of 12 weeks [56]. Following this successful animal study, a clinical randomized controlled trial was initiated (Venous External Stenting Trial, VEST Trial). Nitecki et al. (2017) evaluated a novel braided cobalt-chromium external support for peripheral, i.e., extra-cardiac, vascular reconstructions (FRAME, VGS, Tel Aviv, Israel) in an ovine model (n = 6) of saphenous vein-to-carotid artery bilateral, case-controlled interposition grafting. All six supported and six unsupported grafts remained patent at 12–14 weeks. The diameter of supported veins was unchanged, as opposed to unsupported grafts that significantly dilated, elicited greater lumen irregularity (as shown by the angiographic coefficient of variance), and also developed significantly more intimal hyperplasia [7].

### 4.4. Existing Clinical Data

#### 4.4.1. Coronary Artery Bypass Grafting

First-generation external stents have given disappointing results, e.g., in 2007 with the Extent device (Vascutek, Ltd., Inchinnan, Scotland) that was a knitted Dacron tube reinforced with polytetrafluoroethylene ribs [57]. In 2015, studies with eSVS mesh (nitinol knit, Kipsbay Medical, Inc., Minneapolis, USA) also reported discouraging outcomes [58,59]. Nevertheless, new technologies have emerged, and new trials have been conducted.

Current-generation VEST external support is a cobalt-chrome braid with axial plasticity and radial elasticity. It is manufactured by the same company as FRAME (VGS Ltd., Tel Aviv, Israel); however, VEST is intended for use in heart bypass surgery. In 2015, Taggart et al. first reported a positive effect of VEST on suppressing NIH one year after CABG [60]. A recently published (2023) meta-analysis of three randomized controlled trials (the VEST I trial [60], the VEST III trial [61], and the VEST Pivotal trial [62]) performed between 2011 and 2020, including 437 patients, summarized the current available evidence [9]. Each patient received one stented and one or more non-stented control bypass grafts to obtain a within-patient comparison. VEST did not seem to reduce the incidence of graft failure at short-term follow-up of 1–2 years, although it was associated with significant attenuation of graft nonuniformity or ectasia (as assessed by angiography) and significant reduction of intimal hyperplasia area or thickness (as assessed by intravascular ultrasound). Thus, all risk factors for the development of vein graft atherosclerosis were attenuated. A follow-up of 4.5 years (“the VEST IV trial”) has been reported in 21 patients from the VEST I trial; external stenting significantly reduced diffuse intimal hyperplasia and the development of lumen irregularities; however, graft failure rates were still comparable between stented and non-stented groups [63]. Other meta-analyses [64,65] and a review [13] have reported similar conclusions. Larger trials with longer follow-ups are warranted to determine whether the positive remodeling effects might translate into clinical benefits [6,9].

#### 4.4.2. Peripheral Vascular Surgery

External reinforcements have been used for decades to cover varicose veins used as grafts in peripheral (i.e., extra-cardiac) vascular surgery, most commonly in the lower extremities, thus preventing the development of both graft aneurysm and stenosis. Neointimal hyperplasia is actually more commonly formed in dilated grafts [66,67]. In a prospective multicenter study involving 50 patients and using polyester (polyethylene terephthalate, PET) mesh (ProVena, BBraun, Aesculap, Melsungen, Germany), an acceptable 6-month primary patency rate of 82.3% was achieved with no occurrence of device infection. The indications for infra-inguinal bypass in this study included critical limb ischemia, severe claudication, or popliteal aneurysm. The use of external scaffolding was indicated for varicosity or ectasia of the vein graft or the use of spliced vein grafts with segments of widely differing diameters [66]. In another study involving 21 patients undergoing infra-inguinal bypass surgery with suboptimal, i.e., varicose vein grafts covered with ProVena mesh, the primary patency at 24 months was 57.1%, similar to that of unmeshed, i.e., normal-quality bypass grafts (63.8%). No mesh infection was noted. The authors recommended the use of external mesh in young patients with a long-term bypass patency expectancy to prevent graft dilation [67]. Noteworthily, ProVena mesh is no longer manufactured.

The purpose of external stenting, however, extends to preventing the mid and long-term development of neointimal hyperplasia in grafts of sufficient quality, i.e., non-varicose venous grafts as well [56]. Reports on the use of current-generation external support for normal vein grafts in peripheral vascular surgery are scarce.

Ciftci et al. (2021) performed open surgical repair of popliteal artery aneurysms using FRAME-supported saphenous vein grafts in 12 patients. Eleven subjects received a bypass, and one subject received an interposition graft (mean length 22 ± 5 cm, mean diameter 5 ± 1 mm). Three of the twelve procedures were emergent due to acute limb ischemia. At a mean follow-up of 12 months (range, 7–17 months), the primary patency rate was 100%, there was no change in graft diameters or in the coefficient of variance, and there were no graft revisions, reinterventions, or deep infections. The authors emphasized the potential benefit of using the external stent in patients with aneurysmal arterial disease, as they tend to develop vein graft aneurysms as well [15].

Vigliotti et al. (2022) reported a case of an autologous saphenous vein graft (40–45 cm in length) covered with a FRAME external support to prevent compression in an extra-anatomical position as an axillary-brachial artery bypass around the infected shoulder area. One-year patency was confirmed by a Duplex scan [14].

Vein bypass grafts for lower extremity peripheral arterial occlusive disease are typically longer (40–60 cm) than their coronary counterparts (15 cm) [9] The possible problem with maintaining close contact or conformity along the entire length of the graft may elicit the risk of unprotected segments [43,54]. Furthermore, given the longer grafts’ exposure to bending and the presence of irregular segments that would be excised in coronary grafts, a mildly worse clinical performance of externally stented long infra-inguinal bypass grafts may be encountered [28].

#### 4.4.3. Arterio-Venous Fistulas (AVFs) for Hemodialysis Access

Autologous AVFs for hemodialysis are prone to develop high flow rates and/or aneurysmal dilation. Volume overload correlates with adverse cardiac remodeling in these end-stage kidney failure patients. Steal syndrome and limb ischemia can also occur. Aneurysmorhaphy, with or without external reinforcement, is the surgical treatment method of choice. ProVena mesh has been utilized for external stenting to prevent recurrent high flow after an aneurysmorhaphy procedure [68,69]; however, the results of a randomized AVAH trial are still awaited. Chemla et al. (2016) placed VasQ (Laminate Medical Technologies, Tel Aviv, Israel) metal external support device to improve flow and reduce NIH at the anastomotic site in 20 patients undergoing a brachiocephalic fistula. They reported device safety with high unassisted maturation and patency rates [70].

Matoussevitch et al. (2021) reconstructed and supported 43 high-flow and/or aneurysmal upper arm fistulas in 42 hemodialysis patients using an external stent FRAME FR (VGS). The stent is designed to cover at least 4 cm of the reconstructed fistula and is not intended to cover the cannulation area. Recurrence of high flow (i.e., ≥1500 mL/min) occurred in 16% and 25% of the patients at 6 and 12 months, respectively, while primary patency rates were 86% and 70%, respectively. Only three patients (9%) experienced flow rates exceeding 2000 mL/min and underwent surgical revision to address the recurrent high flow [71]. In contrast, the banding technique is associated with a 52% 12-month-recurrence rate [72]. The authors concluded that the novel external stenting technique is a safe and effective method for reducing and stabilizing flow rates up to 1 year post-operatively. Additionally, their initial learning curve should be considered.

In contrast, Kuemmerli et al. (2020) applied the FRAME device already during the creation of a brachial-basilic upper arm transposition AVF to prevent possible vein dilation. The shunt showed a plateauing flow volume 3 months post-procedurally, illustrating the safety and feasibility of this intervention [73].

### 4.5. Limitations

Our study has several limitations. First, the number of grafts supported with the FRAME device was limited (n = 4), which likely contributed to the marginally significant differences in patency rates and blood flows between groups. The control group had a slightly higher number of grafts (n = 7); however, their mean blood flow post-implantation was significantly lower than that in native carotid arteries. This drop in flow volume was insignificant in the FRAME-supported grafts. The low post-implantation flow is typically attributed to vasospasm of the adjacent carotid artery as long as technical errors are excluded. Nonetheless, we can speculate that the quality or diameter of the control grafts (Pig 2 left and Pig 5 right in particular) could have been suboptimal, potentially compromising their outcomes.

Second, we lack data on graft patency rates and the extent of NIH beyond one month. In a pig model, maximal NIH thickness has been observed at one month, but it can continue to increase over a six-month period [3].

Next, our use of end-to-end interposition grafting, while common in animal models for its simplicity and reproducibility, differs from the more frequent use of end-to-side bypass grafting in actual clinical practice. Angelini et al. reported similar turbulent flow in proximal anastomoses but significantly less turbulent flow in midpoints and distal anastomoses in porcine end-to-end vein-to-artery grafting compared to end-to-side bypass grafting [37]. It is important to note that variations in the angle of beveling in our end-to-end anastomoses occurred (see Table 1) despite all implantations being performed by a single surgeon (J. Ch.).

We acknowledge that our model of internal jugular vein-to-artery grafting may have somewhat less clinical relevance compared to conventional models using saphenous vein grafts, such as those in pigs [46,51], sheep [7,56], or baboons [22,42], since the saphenous vein is commonly used in clinical practice. However, saphenous veins exhibit physiological differences in bipeds and other species, which can limit the applicability of various experimental models [74]. Furthermore, jugular veins engrafted in carotid arteries have previously been utilized in research on vein graft disease in mice [75], rabbits [76,77], and pigs [29,30,31,32,33,34]. These grafts have also been used in studies involving external stenting in animal models (excluding pigs), namely rabbits [45,78,79], dogs [44,80], and sheep [41,81]. Interestingly, Angelini et al. (1990) initially used IJVs as experimental grafts in pig carotid arteries but had to abandon them due to their larger diameters and early ruptures, opting instead for saphenous veins [37]. We speculate that they utilized external rather than internal jugular veins [17]. In contrast, we did not encounter such adverse events, and IJVs were well-matched in diameter with porcine carotid arteries [17,34].

Further, our methodology, which involves placing explanted specimens in warm saline followed by formaldehyde immersion fixation, may have resulted in natural recoil of non-pressurized vessels, potentially leading to tissue analysis in a slightly shrunken state. Most studies have used (perfusion) fixation at arterial pressures, such as 80 mmHg [37], 100 mmHg [38,56], or even systolic 120 mmHg [7,28] to better preserve in vivo dimensions, while others did not [82]. Our group comparisons, however, remain valid, as all samples underwent identical processing. Though speculative, the presence of the healed metal mesh may have provided some protection against sample shrinkage, or conversely, the mesh itself could have exerted a recoil force [22].

We focused exclusively on measuring neo-intimal parameters, in contrast to other studies that also assessed parameters of the medial layer [34,56], the intima-medial thickness [34,38,61], and the adventitia [43]. It is noteworthy that, unlike neointima formation, the preservation of functional smooth muscle mass in the medial layer is a desired outcome when applying an external mesh [43].

Then, only two of our animals (Pigs 3 and 4) received both stented and non-stented grafts as part of a within-subject control strategy. This approach, employed in both preclinical [7,21,51,56] and clinical trials [9], eliminates subject-related factors that might otherwise influence the extent of vein graft disease. In these two animals, however, the right vs. left carotid artery was not randomized for the application of the tested device as opposed to other studies [7,56]. Furthermore, the vein grafts for the right and left sides were specifically taken from their respective sides, thus avoiding the use of vein segments with similar quality from the same vein for implantation. This approach may have influenced the vein remodeling process following implantation [7].

After that, we chose to use female pigs exclusively due to their docile nature and slower weight gain over the study period compared to male pigs. As a result, our experimental findings should not be generalized to both sexes. It is worth noting that many prior studies did not specify the sex of their experimental animals [22,38,43,48].

Finally, it is essential to recognize that our findings pertain to grafts of shorter lengths placed in healthy juvenile animals. These results cannot be directly extrapolated to clinical scenarios involving longer grafts in elderly patients with cardiovascular diseases [55].

### 4.6. Clinical Implications and Future Directions

The results of our study do not have a straightforward clinical impact as the FRAME mesh has already received approval for clinical use in peripheral vascular surgery [18]. However, our study demonstrated the efficacy of FRAME support in our specific animal model, i.e., porcine internal jugular vein-to-carotid artery interposition grafting. Additionally, the study provided us with valuable experience in handling the device before proceeding to clinical use.

Many questions regarding the optimal mesh material, porosity, potential degradation kinetics, and design (such as loose-fitting vs. constrictive, permanent vs. bioabsorbable, or braided vs. knitted) remain unanswered [11,43]. Nonetheless, metal meshes are known to elicit hardly any inflammatory reaction [7,43] and offer protection to vein grafts not only against over-distention and neointima formation [7] but also against external compression and deformation [43].

It is crucial to emphasize that external stenting is not intended to prevent short-term graft failures but rather to mitigate neointimal hyperplasia and superimposed atherosclerosis in the long term [62]. Vein graft failure is a complex issue with multiple contributing factors, and no single treatment is likely to provide a complete solution [11]. Factors such as graft selection, meticulous surgical techniques for vein harvesting, manipulation, preservation, grafting configuration, and anastomosing, along with optimal medical therapy, remain essential for graft function and cannot be replaced by a device-oriented approach [83]. Notably, despite being an innovative and simple device, the mesh incurs additional costs in bypass procedures [84].

Possible future directions may involve combining external support with other interventions for vein graft disease, such as pharmacological treatment [3], gene delivery [85,86], or tissue-engineered vascular grafts.

## 5. Conclusions

In our preclinical model, using an external mesh FRAME enhanced the patency rate of porcine autologous internal jugular vein-to-carotid artery interposition grafts at one month. Upon explant angiography, grafts supported by the FRAME exhibited significantly fewer stenotic areas compared to unsupported grafts. The formation of neointimal hyperplasia was substantially reduced in the FRAME-stented grafts, particularly in the mid portions. Notably, the thickness of the midgraft neointima in the FRAME-supported grafts was half that of their non-supported counterparts.

## Figures and Tables

**Table 1:** List of FRAME-supported and control internal jugular vein interposition grafts implanted in porcine carotid arteries. Beveling refers to anastomosis beveling.

				Right Carotid Artery								Left Carotid Artery							
		Protocol #		Group		Graft		Beveling		Length (cm)		Group		Graft		Beveling		Length (cm)	
Pig 1		50		FRAME		IJV right		No		5.0		FRAME		IJV right		No		3.5	
Pig 2		56		Control		IJV right		Yes		4.0		Control		IJV right		Yes		2.5	
Pig 3		60		FRAME		IJV right		Yes		5.0		Control		IJV left		Yes		5.0	
Pig 4		61		FRAME		IJV right		Yes		5.0		Control		IJV left		Yes		5.0	
Pig 5		20		Control		IJV right		No		2.5		N/A							
Pig 6		29		Control		IJV right		No		4.0		N/A							
Pig 7		48		Control		IJV left		No		4.0		N/A							
					FRAME					Control									
Mean ± SD					4.6 ± 0.8 cm (n = 4)					3.8 ± 1.1 cm (n = 7)					n.s. *p* = 0.227				

IJV—internal jugular vein, N/A—not applicable SD—standard deviation, n.s.—non-significant, #—number.

**Table 2:** Flowmetry results in FRAME-supported and control groups of autologous internal jugular vein interposition graft in porcine carotid arteries at three time points: native carotid artery prior to implantation, after graft implantation, and at explantation (1 month post implantation).

FRAME	Pre-Implantation		Post-Implantation		Explantation 1 M	
	Blood Flow (mL/min)	MAP (mmHg)	Blood Flow (mL/min)	MAP (mmHg)	Blood Flow (mL/min)	MAP (mmHg)
Pig 1 right	85	67	100	72	85	67
Pig 1 left	185	77	220	84	185	77
Pig 3 right	120	76	95	76	120	76
Pig 4 right	190	69	250	67	190	69
Mean ± SD	228 ± 51	73 ± 8	166 ± 80	75 ± 7	145 ± 51	72 ± 5
ANOVA *p* = 0.208	n.s.		n.s.		n.s.	
ANOVA *p* = 0.845		n.s.		n.s.		n.s.
**Control**						
Pig 2 right	160	65	95	56	230	67
Pig 2 left	450	65	60	58	0	70
Pig 3 left	310	88	190	71	22	80
Pig 4 left	320	77	130	68	0	73
Pig 5 right	400	66	20	60	0	82
Pig 6 right	160	56	75	51	70	74
Pig 7 right	270	68	90	89	0	80
Mean ± SD	296 ± 110	69 ± 10	94 ± 54	65 ± 13	46 ± 85	75 ± 6
ANOVA *p* = 0.0001			* vs. Pre-impl.		* vs. Pre-impl.	
ANOVA *p* = 0.177		n.s.		n.s.		n.s.
**FRAME vs. Control**	*p* = 0.280	*p* = 0.601	*p* = 0.107	*p* = 0.186	*p* = 0.066	*p* = 0.419

MAP—mean arterial pressure, SD—standard deviation, M—month, n.s.—non-significant, vs. versus, * statistically significant difference.

**Table 3:** Quantitative angiography results in a tabular heat map. Degrees of stenosis in proximal anastomoses, graft body, and distal anastomoses are given as means from three measurements (anterior-posterior, right lateral, and left lateral (30° angles) projections), calculated according to the North American Symptomatic Carotid Endarterectomy Trial (NASCET) formula (see Methods). Green color indicates no or mild stenosis (≤40%), while yellow and red indicate moderate (41–60%) and severe stenosis/occlusion (>60%), respectively. We considered the left-sided graft in Pig 4 occluded based on zero flow reading. There are significantly more green areas in the FRAME group (9/12) than in the control group (3/21, *p* = 0.0009).

FRAME	Proximal Anastomosis	Graft Body	Distal Anastomosis
Pig 1 right	Severe stenosis 68%	No stenosis *	Mild stenosis 36%
Pig 1 left	Moderate stenosis 60%	No stenosis	Mild stenosis 14%
Pig 3 right	Severe stenosis 66%	No stenosis *	Mild stenosis 40%
Pig 4 right	Mild stenosis 37%	No stenosis *	Mild stenosis 11%
**Control**			
Pig 2 right	Moderate stenosis 56%	No stenosis	Mild stenosis 29%
Pig 2 left	Occlusion	Occlusion	Occlusion
Pig 3 left	Severe stenosis 76%	Mild long stenosis 25%	Medium stenosis 41%
Pig 4 left	Severe stenosis 73%	Long near occlusion 84%	Near occlusion 83%
Pig 5 right	Occlusion	Occlusion	Occlusion
Pig 6 right	Moderate stenosis 47%	Moderate long stenosis 40%	Moderate stenosis 52%
Pig 7 right	Occlusion	Occlusion	Occlusion

* Mild eccentric semicircular non-stenotic neo-intimal hyperplasia on cross-sections.

**Table 4:** Area and thickness of neo-intimal hyperplasia (NIH) in FRAME-supported and control internal jugular vein interposition grafts in porcine carotid arteries at one month post implantation. Data are presented as means ± standard errors of NIH area and thickness computed from a digital analysis of macrographs of serial cross-sections along the grafts. Thickness was calculated as the NIH area divided by the original lumen circumference.

Neointimal Area (mm^2^)	Proximal Anastomosis	Midgraft	Prox. Anastomosis and Midgraft
Control	6.94 ± 1.10	6.27 ± 0.64	6.38 ± 0.56
FRAME	5.79 ± 1.34	3.73 ± 0.64	4.53 ± 0.68
Reduction (%)	16.6%	40.5%	29%
*t*-test	n.s. *p* = 0.558	* *p* = 0.022	* *p* = 0.044
**Neointimal Thickness (μm^2^/μm)**			
Control	587 ± 52	518 ± 36	530 ± 32
FRAME	480 ± 95	258 ± 49	344 ± 53
Reduction (%)	18.2%	50.2%	35.1%
*t*-test	n.s. *p* = 0.401	* *p* = 0.0002	* *p* = 0.002

n.s.—non-significant, * significant.

**Table 5:** Published studies using the internal jugular vein-to-carotid artery model in pigs. The conventional vein graft harvest involves a technique as opposed to a no-touch technique.

Study	Objective	Animal Model	Graft Harvest	Configuration	Heparin	Antiplatelet	Period	Patency
Chen et al. 1994 [29]	Vein graft gene transfer (iNOS)	Farm pig	N/R, prob. conventional	Interposition end-to-end	300 IU/kg	Aspirin 150 mg (3 ds bef.)	3 ds	100% (8/8)
Kibbe et al., 2001 [34]	Vein graft gene transfer (VCAM)	Domestic pig	N/R, prob. conventional	Interposition end-to-end	100 IU/kg	N/R	21 ds	100% (8/8)
Bartels et al., 2003 [30]	Vein graft brachytherapy, control group	Hyperchol. Landrace pig	N/R, prob. conventional	Bypass end-to-side	N/R	Aspirin 100 mg (post-op.)	4 wks	87.5% (14/16)
Jevon et al., 2011 [31]	Vein graft disease study	Inbred Landrace pig	N/R, prob. conventional	Interposition end-to-end	1000 IU/kg	N/R	4 wks	100% (4/4)
Quint et al., 2011 [32]	Tissue engineering, control group	Yorkshire pig	N/R, prob. conventional	Bypass end-to-side	100 IU/kg	Aspirin 5 mg/kg + clopidogrel 1 mg/kg, (1 d bef.)	30 ds	37.5% (3/8)
Thim et al., 2012 [33]	Vein graft disease study	Hyperchol. minipig	Conventional, no distension	Interposition end-to-end not beveled	Yes, dose N/R	Aspirin 150 mg (post-op)	12–14 wks	88.9% (8/9)
Our study	External stenting, control group	Domestic pig	Conventional, gentle distension	Interposition end-to-end	200 IU/kg	Aspirin 100 mg (1 d bef.)	4 wks	42.9% (3/7)

Abbreviations: before surgery and post-operatively (bef.), day (d), days (ds), hypercholesterolemic (hyperchol.), inducible nitric oxide synthase (iNOS), not reported (N/R), post-operatively (post-op.), probably (prob.), vascular cell adhesion molecule (VCAM), weeks (wks).

## Data Availability

The original contributions presented in the study are included in the article; further inquiries can be directed to the corresponding author.
